# MRPS17 promotes invasion and metastasis through PI3K/AKT signal pathway and could be potential prognostic marker for gastric cancer

**DOI:** 10.7150/jca.55719

**Published:** 2021-06-11

**Authors:** Wenjie Zhou, Jun Ouyang, Junqing Li, Fangjie Liu, Tailai An, Lvjia Cheng, Zi Chong Kuo, Changhua Zhang, Yulong He

**Affiliations:** 1 Digestive Disease Center, The Seventh Affiliated Hospital, Sun Yat-sen University, Zhenyuan Road 628, Guangming District, Shenzhen 518000, Guangdong, China.; 2 Department of Gastrointestinal Surgery, The First Affiliated Hospital, Sun Yat-sen University, Zhongshan 2nd Road 58, Yuexiu District, Guangzhou 510080, Guangdong, China.; 3 Department of Pathology, The Seventh Affiliated Hospital, Sun Yat-sen University, Zhenyuan Road 628, Guangming District, Shenzhen 518000, Guangdong, China.; 4 Department of Hematology, The Seventh Affiliated Hospital, Sun Yat-sen University, 58 Zhongshan 2nd Road 58, Yuexiu District, Guangzhou 510080, Guangdong, China.; 5 Scientific Research Centre, The Seventh Affiliated Hospital, Sun Yat-sen University, 628 Zhenyuan Road 628, Guangming District, Shenzhen 518000, Guangdong, China.

**Keywords:** gastric cancer, TCGA, PI3K/AKT signaling pathway, invasion and migration

## Abstract

In this study, the molecular mechanisms through which Mitochondrial Ribosomal Protein S17 (MRPS17) contributes to gastric cancer (GC) and its prognostic significance in GC have been explored. As a protein encoding gene, MRPS17 encodes a 28s proteins belonging the ribosomal protein S17P family. The specific roles and molecular mechanisms of MRPS17 in cancers remain ambiguous. It was revealed by analyzing data from TCGA and GEO that elevated expression of MRPS17 was significantly associated with invasion of GC and poor survival of GC patients. Then through univariate and multivariate Cox regression analyses it was demonstrated that MRPS17 an independent prognostic factor for GC patients (*P*<0.001). It was demonstrated by differentially expressed gene analysis and functional enrichment analysis that MPRS17 is related to PI3K/AKT pathway and Cell adhesion molecules (CAMs), while its function is mediated by collagen-containing extracellular matrix and receptor ligand/regulator activity. Then it was proven by in-vitro experiments that knocking down of MRPS17 gene in AGS and SGC7901 cells would significantly inhibit proliferation and invasion capability of these cells. Furthermore, it was revealed by cell immunofluorescence assay that as a ribosomalprotein, MRPS17 was mainly distributed in the cytoplasmic surface of cell membrane. Additionally, activation of PI3K/AKT pathway is responsible for malignant progression of glioma that was promoted by MRPS17.

In conclusion, it was revealed in the present study that MRPS17 promoted invasion and metastasis of GC and potential molecular mechanisms through which it exerted its influences on GC were explored, suggesting its potential as a novel prognostic biomarker for GC.

## Introduction

Gastric cancer (GC) is one of the most commonly diagnosed malignant tumors and curative surgery remains the treatment of choice for non-metastatic GC 1. With the application of multidisciplinary teamwork mode, the 5-year survival rate of patients diagnosed with early gastric cancer can reach 95% 2. However, due to the elusive nature of GC and difficulties in diagnosing early GC, most patients are at an advanced stage when the diagnosis is made and curative surgery is not possible 3. For patients with advanced GC, radiotherapy, chemotherapy, targeted therapy and immunotherapy (one modality or more) are usually applied. Thus, it is of significant importance for us to develop novel treatment alternatives and explore new prognostic markers to improve the prognosis of the patients that have been diagnosed with advanced GC 4, 5.

Encoded by nuclear genes, mammalian mitochondrial ribosomal proteins are mainly involved in synthesis of mitochondrial proteins. Mitochondrial ribosomes are comprised of a small subunit (28S) and a large subunit (39S) 6, 7. The protein to rRNA ratio of mitochrondrial ribosomes is 3:4, which is 4:3 in prokaryotic ribosomes. Another aspect that distinguishes prokaryotic ribosomes from mammalian mitoribosomes is that the former ones contain a 5S rRNA 8. Amino acid sequences of mitoribosomal proteins and their biochemical properties vary significantly among different species, which makes it difficult for us to distinguish them easily 9. As a protein encoding gene, MRPS17 encodes a protein (28s) belonging to the ribosomal protein S17P family, which was moderately conserved between human and prokaryotes 10. MRPS17 has been reported to be involved in the pathogenesis of myxosarcoma and phosphoserine phosphatase deficiency. MRPS17-related processes include biogenesis and maintenance of organelles and translation of viral RNA. Varvagiannis et al. 11 reported that a 27-month-old boy showed psychomotor delay and deformity features, mainly manifested as mild facial asymmetry. The case discussed CHCHD2, GBAS, MRPS17, SEPT14 and PSPH may be related to the patient's regarding phenotype, the unbalanced region of the patient's chromosomes also contains three genes that are considered essential to mitochondrial function, namely CHCHD2, GBAS and MRPS17. MRPS17 encodes the mitochondrial ribosomal protein S17, which has an indirect role in the mitochondrial translation system and the mrps17 is necessary for the production of 13 proteins necessary for OxPhos 12. According to Gene Ontology (GO) database, MRPS17-related annotations include rRNA binding and constitution of ribosome. ENSG00000249773 is one important paralog of MRPS17. To investigate the prognostic value and biological function of RNA binding proteins (RBPs) in stomach adenocarcinoma (STAD), we conducted a prognosis of gastric cancer related RBP research 13. Seven RBPs (PTBP1, PPIH, SMAD5, MSI2, RBM15, MRPS17, and ADAT3) were identified to be prognosis-related and adopted to construct a prognostic model. In the study, we found that MRPS17 is strong correlation with prognosis, and there is currently no research on this gene.

In the present study, prognostic significance of MRPS17 and its potential roles in GC were explored. Based on results of analyzing the data from public database, we further evaluated clinical and prognostic significance of MRPS17. Then through KEGG and GO functional enrichment analysis, biological functions of MRPS17 and molecular mechanisms were identified. Afterwards, we performed in-vitro experiments, demonstrating that knocking down of MRPS17 in different GC cell lines could significantly inhibit proliferation and invasion of these cells. Additionally, we verified the associations between MRPS17 and PI3K/AKT signaling pathway, and cell adhesion molecules. Considering all these aforementioned results, we could draw the conclusion that MRPS17 could be utilized as a novel therapeutic and prognostic biomarker for GC.

## Materials and methods

### Data processing

Relevant RNA-sequencing and clinical data of GC were extracted from the TCGA (https://portal.gdc.cancer.gov/) database. All the downloaded expression profiles were acquired as HT-seq read counts and interpreted according to the Ensembl reference database (http://www.ensembl.org/info/data/ftp/index.html). DEMs (differentially expressed mRNAs) between GC tissues and normal gastric tissues were identified using the “DESeq2” package of R software. mRNAs whose adjusted *P*-values were <0.05 and log2|fold change| values were >1 were defined as DEMs. The present study was performed in compliance with data access rules and publication guidelines of TCGA database. Heatmaps of this study were made visualized using “pheatmap” and “ggplot2” of R software.

### GO enrichment and KEGG pathway analysis

Genes were classified according to the projection at a specific level of GO terms or KEEG pathways by “clusterProfiler” package of R software. Then GO terms and KEGG pathways were performed through a hypergeometric distribution with *P* < 0.05 as the significance threshold.

### Immunohistochemistry

The formalin-fixed and paraffin-embedded sections were deparaffinized with xylene and then rehydrated. Antigen retrieval was performed with Tris/EDTA buffer pH 9.0 for 20 min at 95 ºC in paraffin-embedded tissue sections. The slides were incubated with antibodies against MRPS17 (1:300, Proteintech Group, USA) overnight at 4 °C. The reactivity was detected using Dako EnVision-HRP (Dako). Two pathologists are responsible for reviewing each slide and score independently.

### Cell culture and siRNA transfection

The gastric cancer cell lines used in our study including AGS, SGC7901 were bought from the Shanghai Institute of Cell Biology, Chinese Academy of Sciences (Shanghai, China). After being rewarmed, SGC7901 cell line was cultured on dishes filled with RPMI-1640 (Shanghai XP Biomed Ltd., Shanghai, China), while AGS cell line with DMEM/F-12(HAM) medium (Shanghai XP Biomed Ltd., Shanghai, China). Both the two aforementioned culture media were added with 10% fetal bovine serum (FBS; Sage Creation Science Co., Ltd., Beijing, China) and 1% penicillin and streptomycin solution (Beijing Solarbio Science & Technology Co., Ltd., Beijing, China). These two cell lines were cultured at 37 °C in a 5% CO2-filled atmosphere and were detected with no mycoplasma contamination. Guangzhou RiboBio Co. Ltd. (Guangzhou, China) was responsible for designing and synthesizing GenOFFTM si-h-MRPS17 siRNA and negative control siRNA oligonucleotides, the sequences of which were presented in Supplementary Table 1. Under instructions provided by the manufacturer, we performed the siRNA transfections with 100 nm pooled siRNA and riboFECT^TM^ CP Transfection Kit (Guangzhou RiboBio Co. Ltd., Guangzhou, China).

### RNA extraction and quantitative real-time PCR

RNA fast 2000 Reagent (Fastagen, Shanghai, China) was adopted to extract total RNA from SGC7901 and AGS cells, which was then quantified using the NanoDrop spectrophotometer (Thermo Fisher Scientific, Waltham, MA, USA). Then under the instructions provided by the manufacturer, we reverse-transcribed 1 μg of total RNA using a PrimeScript^TM^ RT Reagent Kit purchased from Takara (Dalian, China). 2 μL of cDNA was put into a 20-μL reaction tube to be utilized in quantitative real-time PCR performed by CFX96 Real-Time PCR Detection System (Bio-Rad, Shanghai, China). The PCR primer sequences used in our study were as follows: GAPDH(F): ACAACTTTGGTATCGTGGAAGG; GAPDH®: GCCATCACGCCACAGTTTC; MRPS17(F): ATGTCCGTAGTTCGCTCATCC; MRPS17®: CCTGGTCACTCTCACTTTAGCA. All the aforementioned reactions were performed in triplicate without any template control used in each run. The expression of each target gene was standardized with GADPH as the endogenous control and the relative target gene level was determined using the 2-ΔΔCT method.

### Transwell assay

24-well Transwell plates with 8-μm sized pores (Corning) were used to perform Transwell assays. After MRPS17 siRNA or control siRNA was successfully transfected into AGS and SGC7901 cell lines, 5×10^4^ cells of both cell lines cultured in serum-free medium were planted into the upper chamber with the lower chamber filled with 800 µL 10% FBS-supplemented medium. After being incubated for 24 hours, the chambers were then washed with phosphate-buffered saline (PBS). Afterwards, cells that were still located within the upper chamber were wiped off with cotton swabs while those having migrated into the lower chamber were chemically fixed with methanol, stained with Giemsa. Then, the stained cells were visualized and photographed using a microscope (Olympus CKX53). The acquired images were then processed using ImageView (X64, version 4.7.14963). For invasive assays, the procedures were basically the same except for 10% Matrigel (BD Biosciences) precoated within upper chamber and doubled number of seeded cells.

### Western blotting

After being washed using PBS, the cells were lysed with RIPA (radioimmunoprecipitation assay) solution supplemeted with protease inhibitor. The proteins obtained from the lysed GC cells were then quantified using a bicinchoninic acid protein assay (BCA) kit, after which 20 μg of total protein was separated on 8% or 10% sodium dodecyl sulfate polyacrylamide gel (SDS-PAGE) under electric field. The separated proteins were transferred onto a polyvinylidene difluoride (PVDF) membrane (Millipore, USA), which was then blocked with 5% bovine serum albumin (BSA) solution for one hour and incubated with primary antibodies against GAPDH, AKT, P-AKT and MRPS17 (1:1000, Proteintech Group, USA) overnight at 4 °C. On the second day, the membrane was incubated with secondary antibodies (1:2500) at room temperature for one hour after three times of washes with TBST (Tris-buffered saline-Tween). After being washed for another three times, bands of antibody-conjugated proteins were visualized via a ChemiDoc^TM^ MP Imaging system (Bio-Rad Laboratories) with GADPH as the internal control.

### Statistical analysis

Wilcox test was adopted to compare differences between two groups while comparisons among multiple groups were made by Kruskal test (two-tailed test). These tests adopted to verify the associations between MRPS17 expression and clinicopathological variables such as age, gender, TNM stage, and so on. They were used to compare the associations of continuous and categorical variables like age, gender. Survival curves were plotted according to the Kaplan-Meier method and compared by log-rank tests. All quantitative variables in the present study were demonstrated as mean ± standard deviation. Statistical softwares involved in our study included GraphPad Prism 5.0 (GraphPad, La Jolla, CA, USA), SPSS software (version 23.0) and R software (version 3.6.2). P values that were <0.05 were deemed as statistically significant unless specially indicated.

## Results

### Expression of MRPS17 in gastric cancer

**Figure 1:**
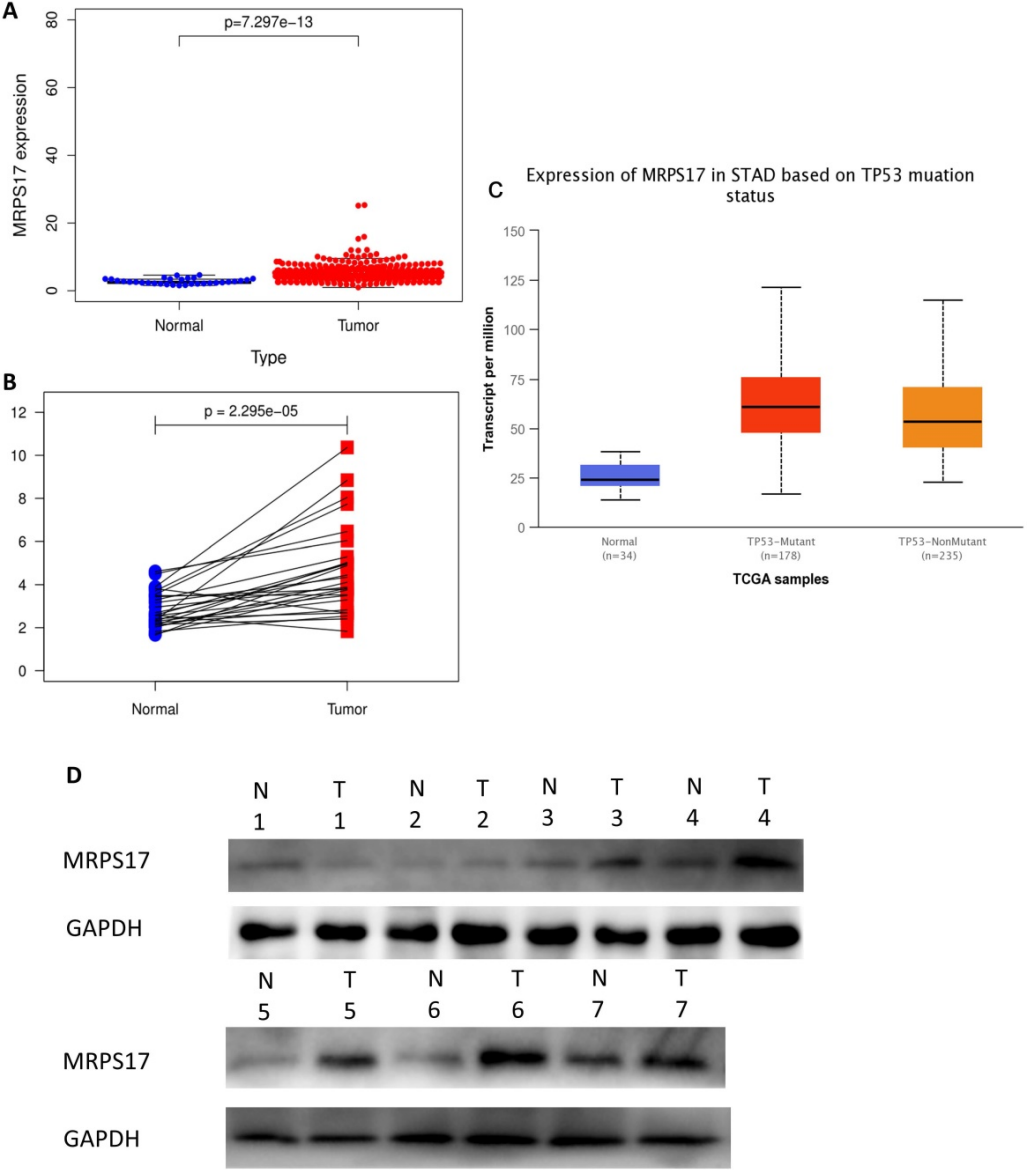

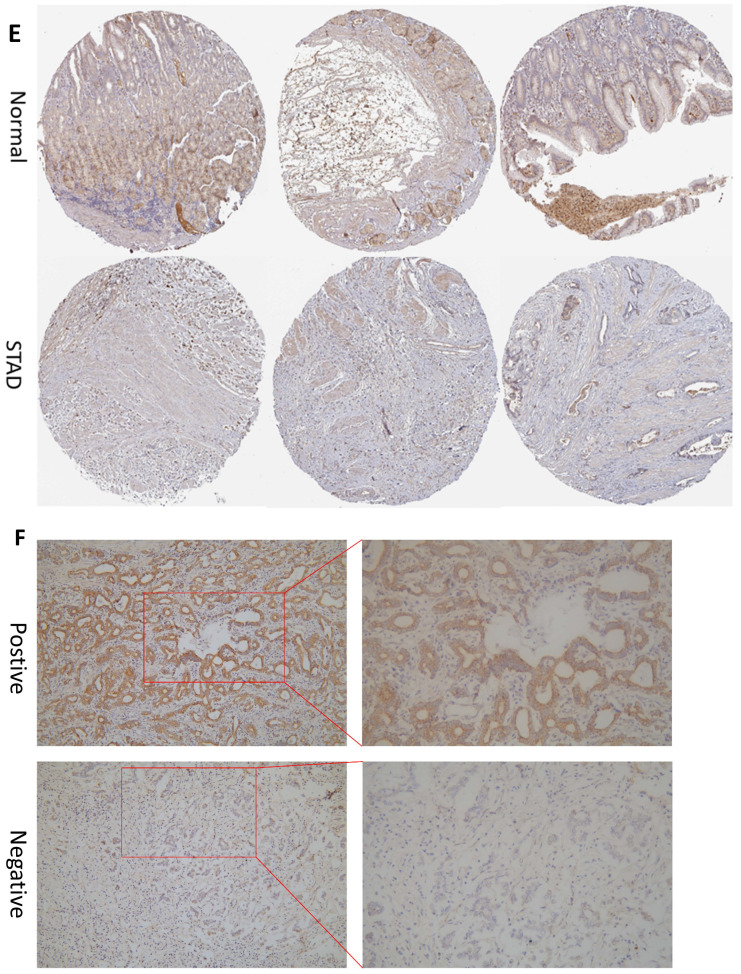
Analysis of the MRPS17 expression levels in normal tissues and GC tissues. (A) Normal tissue and tumor tissue (B) paired normal tissue and tumor tissue. (C) Normal tissue and tumor tissue expressing with TP53. (D) MRPS17 protein expression in normal tissue and tumor tissue paired by our center. (E) The expression of MRPS17 in normal gastric tissues and GC tissues in the HPA database. (F) Evaluation of positive/negative expression of MRPS17 in gastric cancer patients in the database of our center.

In order to determine if the MRPS17 is different between gastric cancer and normal tissue, we first analyzed the gene sequencing data results of all gastric cancer tumor tissue specimens in the TCGA database. In the data sample set of 413 people gastric cancer samples and 34 normal gastric tissues, the expression of MRPS17 in gastric cancer increased significantly (Figure 1A-B). At the same time, we found that the expression level of MRPS17 increased significantly in TP53-Mutant patients in subgroup analysis expressed based on TP53 mutation status (Figure 1C). After that, we collected 7 pairs of gastric cancer and normal gastric tissues (more than 5CM from the edge of the tumor) clinical specimens from our center, and extracted the total protein of the cancer and normal gastric tissues, further verified the expression level of MRPS17 through the WesternBlot test (Figure 1D). As we can see from the figure, the expression level of MRPS17 in cancer tissue (T) is generally higher than that in normal tissue (N). We further used tissue samples from the HPA database to observe the public results of MRPS17 immunohistochemical detection related experiments and found that MRPS17 is highly expressed in gastric cancer tissues (Figure 1E). After reviewing the medical records of gastric cancer patients in our center, we randomly selected and grouped 100 gastric cancer patients for further immunohistochemical testing and scoring (Figure 1F).

### Prognostic correlation analysis

**Figure 2:**
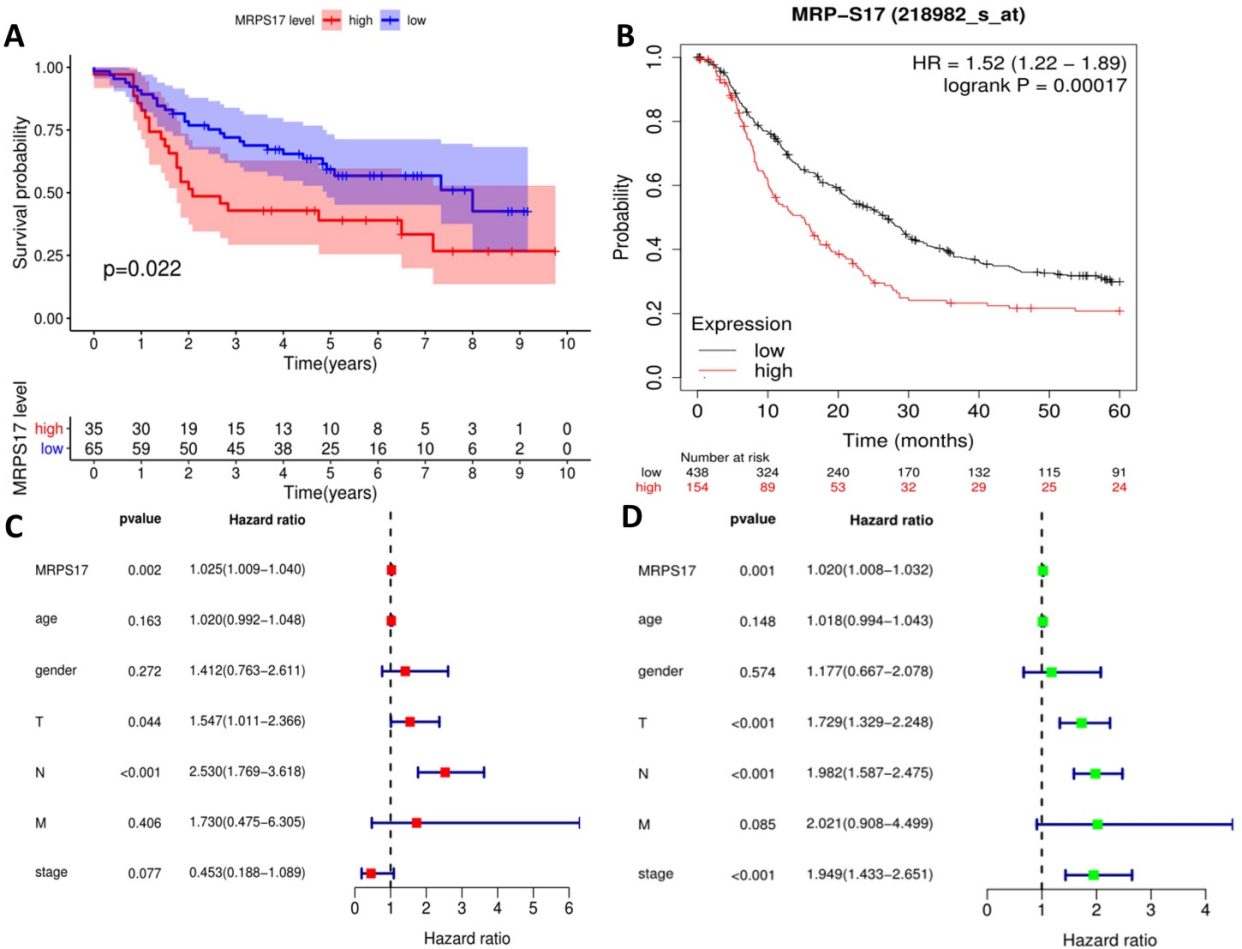

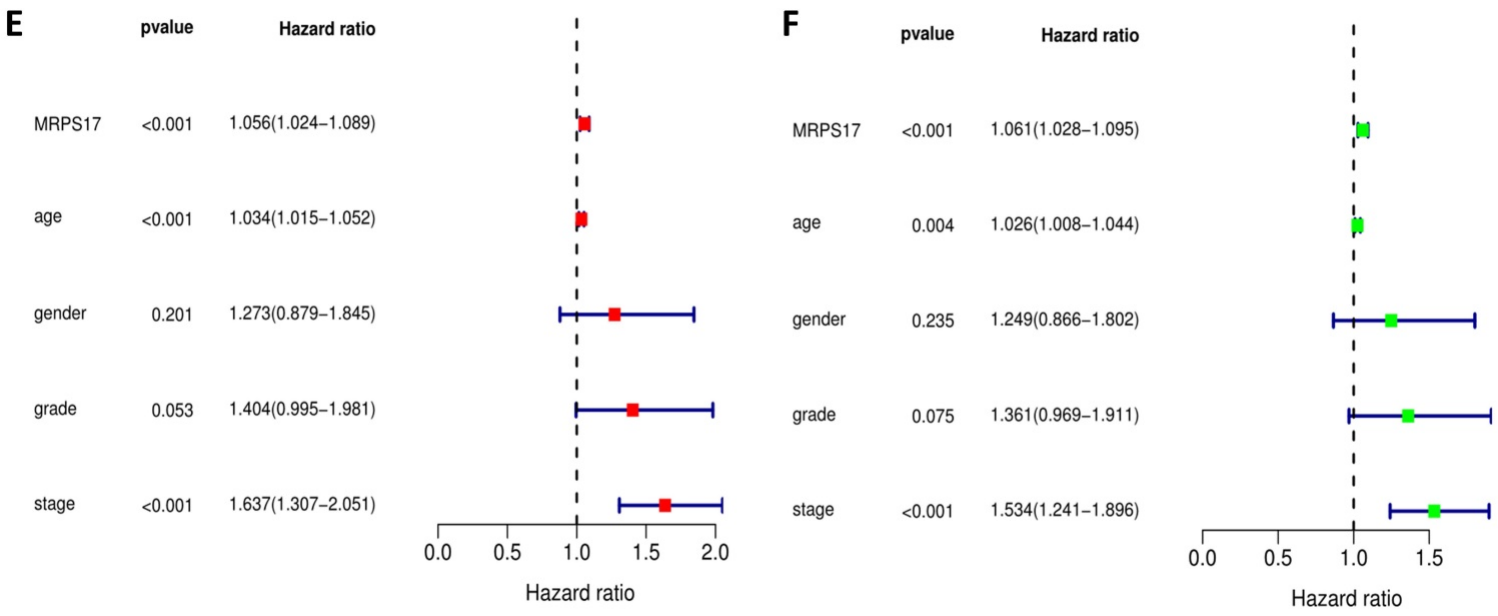
** Prognosis analysis of MRPS17.** (A) The patient's survival prognosis in the database of our center. (B) Kaplan-Meier survival curves for MRPS17 in GEO database. The tick-marks on the curve represent the censored subjects. The number of patients at risk is listed below the curve. Univariate (green) and multivariate (red) COX regression analysis of different clinical parameters in our center (C-D) and TCGA (E-F) database.

We further sorted out and summarized the clinics of 100 patients in our center (Supplementary Table 2). We grouped MRPS17 patients according to the results of two independent pathologists with the scores of the immunization group then calculated the prognosis of the patients. According to our results, the prognosis of MRPS17 positive patients was significantly worse than that of negative patients (Figure 2A, *p*=0.022). At the same time, we used the data of GEO database to perform Kaplan-Meier Plotter survival analysis for further verification (Figure 2B, *p*<0.01). After that, we further collated the clinical data in the TCGA database (Supplementary Table 3), which those patients with incomplete clinical and prognostic data are eliminated, and found that that whether it is in our center's database (Figure 2C-D) or TCGA (Figure 2E-F), the expression of MRPS17 is an independent prognosis whether in the multivariate (red) and univariate (green) COX regression.

### Clinical relevance analysis of MRPS17

After that, we analyze the correlation between the main tumor indicators (age, gender, tumor invasion depth, metastasis, etc.), and further divide the patients into T1~2 groups and T3~4 groups according to the depth of gastric cancer invasion. In the correlation calculation, the median is used as the cutoff value. Correlation analysis showed that patients diagnosed with T3-4 gastric cancer had high expression of MRPS17 (Figure 8C-D, P<0.001), suggesting that MRPS17 is more likely to be highly expressed in advanced or aggressive gastric cancer.

### Biological function and signaling pathway regulation

**Figure 4:**
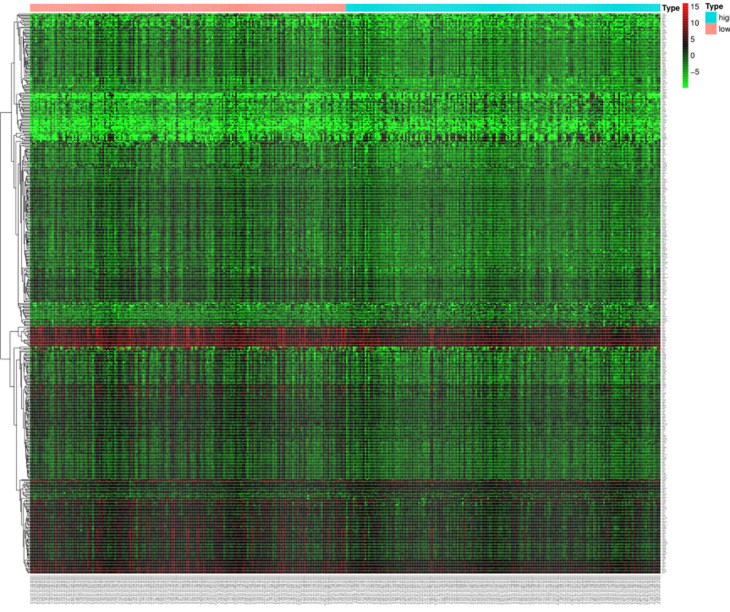
Heatmap of the 474 differentially expressed genes in GC patients according to TCGA (logFC>1, *P* < 0.05).

**Figure 5:**
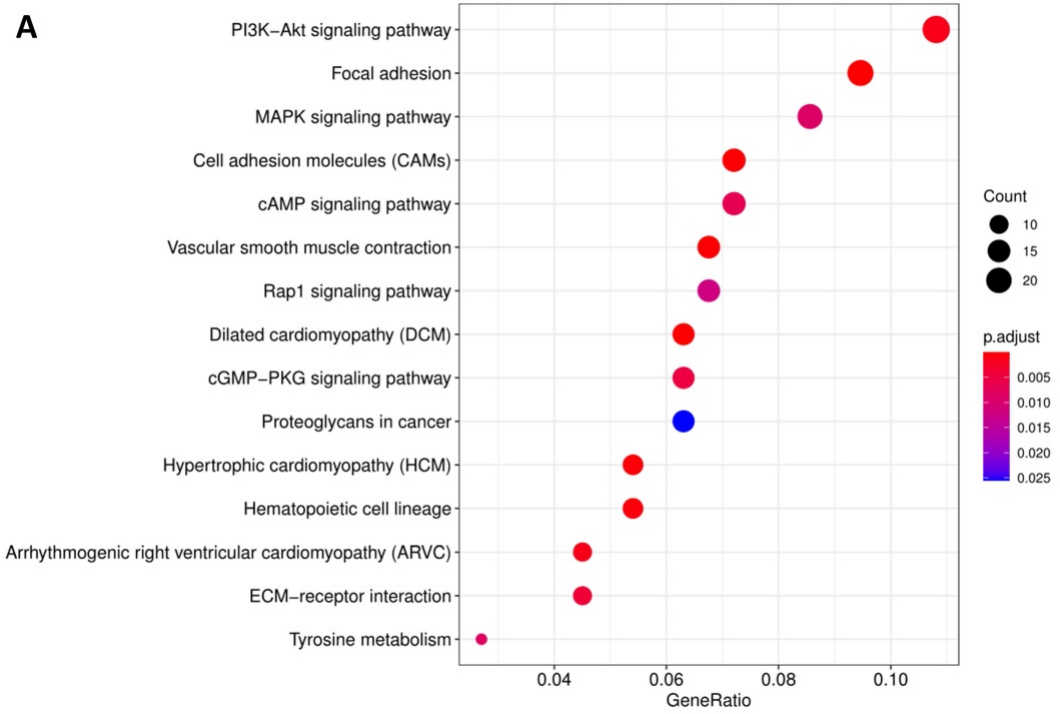

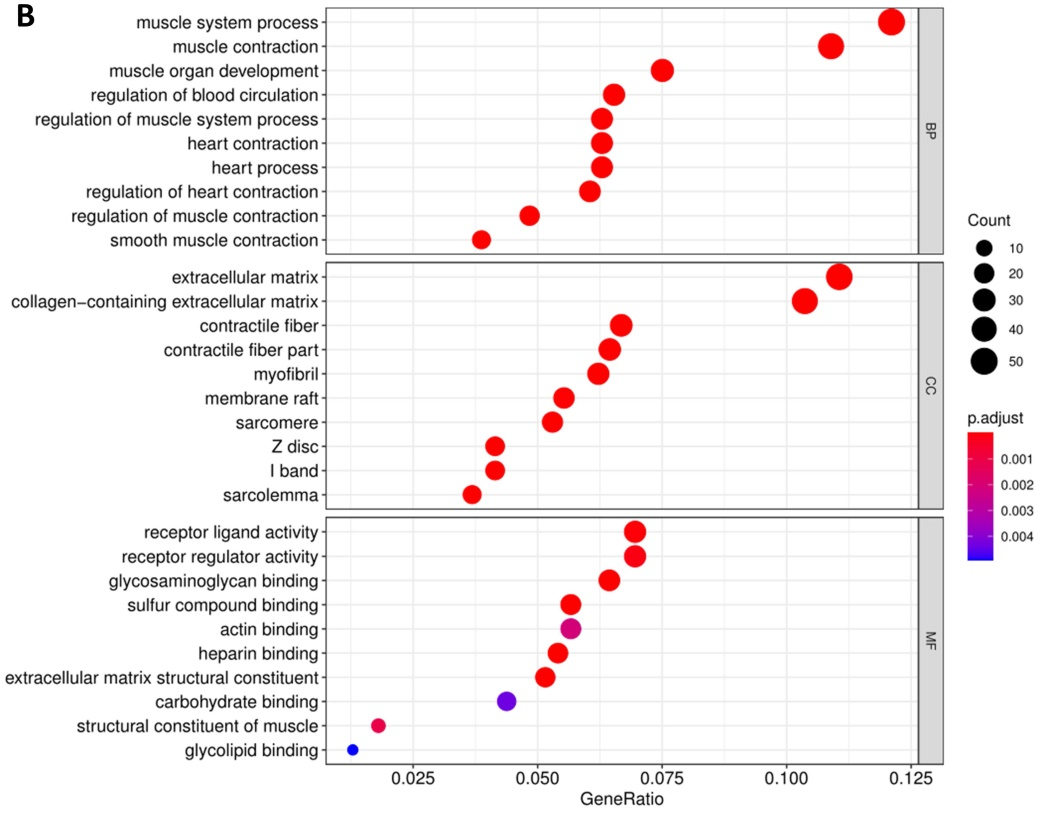
Pathway enrichment of differentially CDMs by GO (A) and KEGG (B) analysis.

In order to further understand the biological function of MRPS17 in gastric cancer, we analyzed data from TCGA-STAD database to determine MRPS17-related genes by genome-wide gene profile, results of which demonstrated that a total of 474 genes were related to MRPS17 expression (logFC > 1, *P* < 0.05; Figure 4). According to the plotted heatmap, these 474 genes were divided into positive or negative correlated ones. Subsequently, it was revealed by GO analysis that genes positively related with MRPS17 expression were mainly involved in multiple biological processed including collagen-containing extracellular matrix and receptor ligand/regulator activity (Figure 5A). Furthermore, by performing KEGG analysis, we revealed that MRPS17-related genes participated in multiple cancer-related signaling pathways (especially in PI3K/AKT) and were significantly correlated with cell adhesion molecules (CAMs) (Figure 5B). Thus, considering all these results, we might hypothesize that MRPS17 could increase proliferation and invasion capability of GC cells through interacting with CAMs and extracellular matrix via regulating PI3K/AKT signaling pathway.

### Expression of MRPS17 in gastric cancer cells

**Figure 6:**
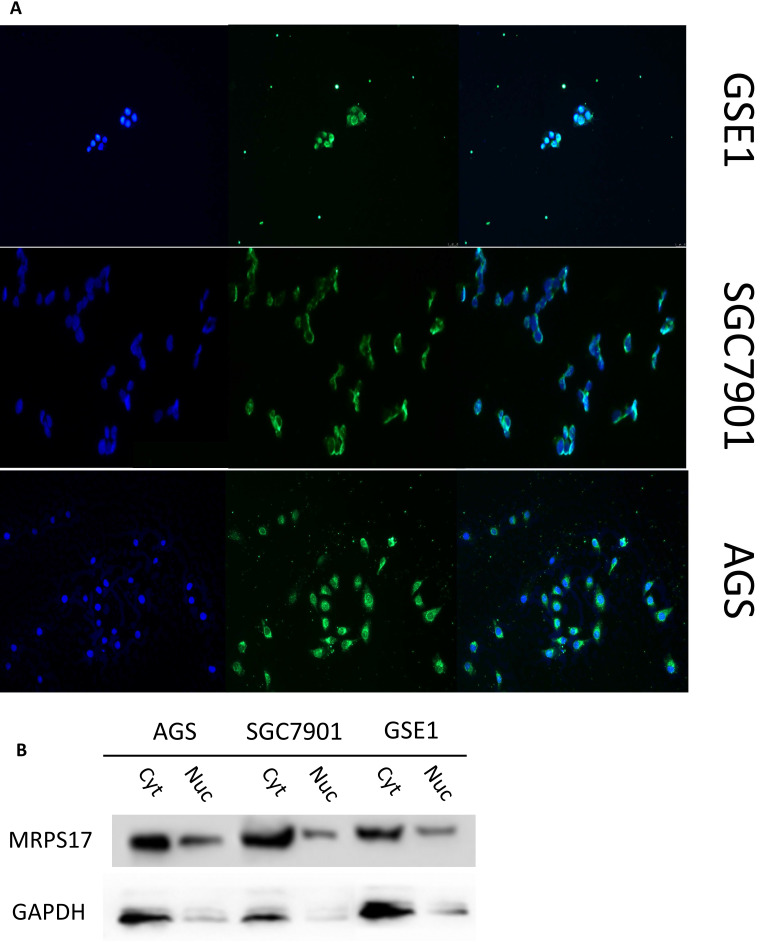
(A) Evaluation of the expression of MRPS17 in normal gastric cell lines GSE1 and gastric cancer cell lines AGS and SGC7901 by immunofluorescence (B) MRPS17 expression in GSE1, AGS and SGC7901 cell lines after nucleocytoplasmic separation.

As a potential RNA binding protein, the specific function and molecular mechanism of MRPS17 in gastric adenocarcinoma have not been fully explored. Therefore, we subsequently performed immunofluorescence analysis with AGS, SGC7901 and GSE1 cell lines, and found that MRPS17 is not only located in the cytoplasm but also significantly located on the nucleus and cell membrane, which may contribute to the interaction of MRPS17 with CAM and extracellular matrix (Figure 6A). At the same time, we performed nucleocytoplasmic separation and protein determination of the corresponding cells. We found that MRPS17 is expressed in both nucleus and cytoplasm, and compared with normal gastric cell lines, MRPS17 has a higher nuclear protein expression in gastric cancer cell lines, suggesting the potentially important role of MRPS17 in the progression of gastric cancer cells (Figure 6B).

### Knocking down MRPS17 inhibits GA cell migration and invasion *in vitro*

**Figure 8:**
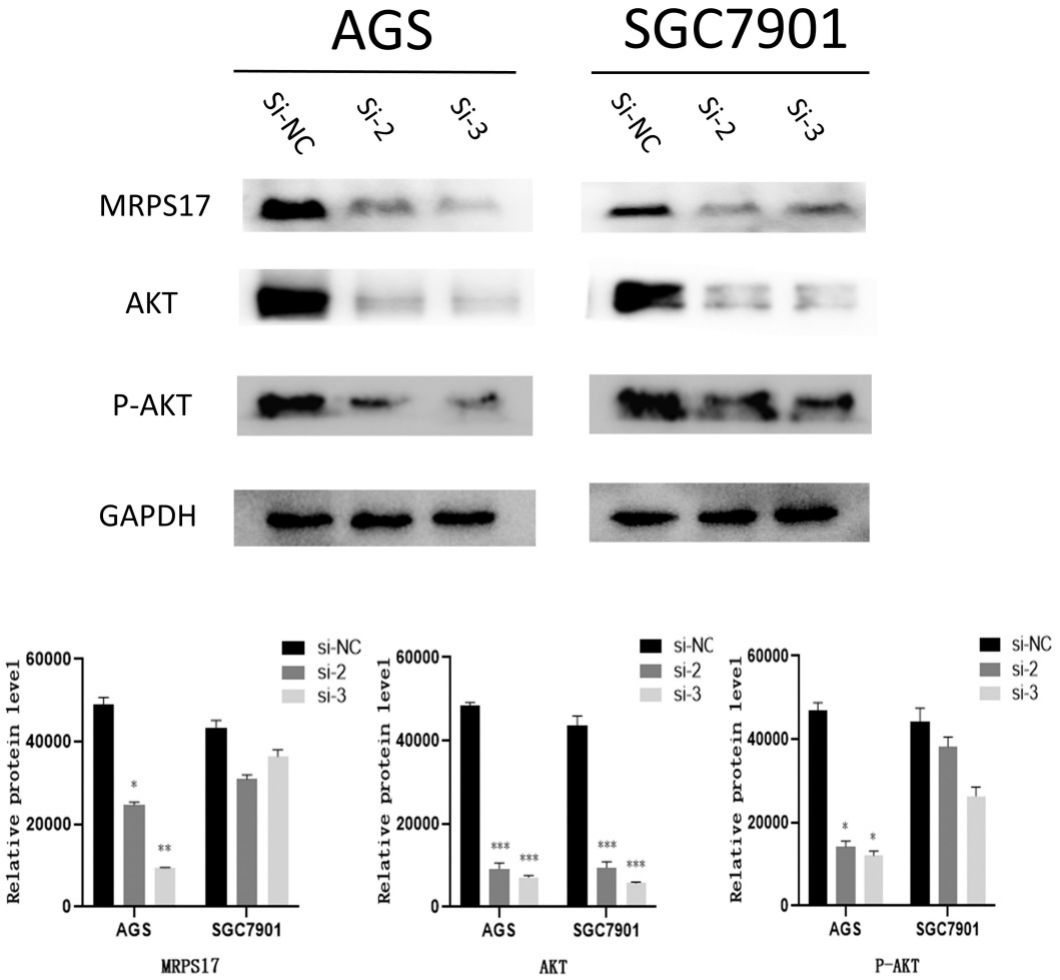
The expression levels of MRPS17, AKT, P-AKT, GAPDH were evaluated by Western blotting after MRPS17 had been knocked down.

With the aim of further verifying the biological functions of MRPS17 in GC, we first evaluated the expression levels of MRPS17 in different GC cell lines by performing QT-PCR, result of which demonstrated that MRPS17 was significantly up-regulated in a few GC cell lines, especially in AGS and SGC7901 (Figure 8A). Compared with AGS or SGC7901 cells that had been transfected with si-NC, those had been transfected with si-1 or si-2 or si-3 had significantly reduced MRPS17 mRNA level, especially in cells transfected with si-2 and si-3 (Figure 8B). Therefore, we chose GC cells that had been transfected with si-2 or si-3 (SGC-7901 or AGS) for subsequent studies. Considering the finding by GO analysis that MRPS17 might participate in cell adhesion and regulation of extracellular matrix, we further assessed the effects of knocking down MRPS17 on migration and invasion capability of GC cells. Matrigel precoated in the upper chamber of the Transwell system was utilized to mimic the extracellular matrix of GC. By Transwell assays, we revealed that knocking down MRPS17 significantly inhibited migration and invasion capability of AGS and SGC7901 cells (Figure 4C and E).

### MRPS17 regulates the malignant behavior of GC through PI3K/AKT signaling pathway

As known to us, abnormal activation of PI3K/AKT signaling pathway has been reported to promote carcinogenesis. Then by KEGG analysis, it was also demonstrated that multiple MRPS17-related genes were influenced by PI3K/AKT signaling pathway (Figure 6B).

Thus, we performed Western blotting analyses to quantify the levels of phosphorylated-AKT (P-AKT) and AKT, results of which demonstrated both levels of AKT and P-AKT significantly reduced after MRPS17 had been knocked down, suggesting that MRPS17 might promote aggressive behavior of GC through regulating the PI3K/AKT signaling pathway (Figure 8).

## Discussion

As one of the most commonly diagnosed malignant tumors worldwide, GC originates from gastric mucosal epithelial cells. Annually, the number of diagnosed GC exceeds 1 million 14. By far, curative surgery remains the treatment of choice for most GC patients despite the progress made in chemotherapy, radiotherapy, targeted therapy and immunotherapy 15. Although quality of life of GC patients have been remarkably improved, prognosis of patients with advanced GC remains quite poor since the five-year survival of these patients is still less than 30% 16. Due to the difficulties in diagnosing early GC, most patients have reached an advanced stage when the diagnosis is initially made and curative surgery is not possible to be performed. For patients with advanced GC, despite the fact that palliative treatments such as radiotherapy, chemotherapy, targeted therapy or immunotherapy are usually applied, their survival is still not satisfying, so making developing new treatment alternatives a crucial task for us.

Mitochondrial ribosomal protein (MRP) is a basic component of mitochondrial complex structure and functional integrity. More than 80 MRPs have been identified in mammals, and 79 MRP gene expression patterns were recorded in mice development and adult organization, which found that these genes always express consistent throughout the early embryos, there is almost no stage or organization specificity 10. Huang et al. 17 have compared the designated position of the MRP gene with candidate regions of human diseases. Defects in the mitochondrial translation device can lead to abnormal phenotypes such as MELAS (mitochondrial myopathy, encephalopathy, lactic acidosis and stroke-like seizures), MERRF (myoclonic epilepsy associated with red fibers) and deafness, MRP defects. It may also lead to mitochondrial dysfunction and related pathological conditions. Of particular interest is that MRP may be related to deafness. In fact, we have found multiple MRP genes in key areas of hereditary hearing loss. Among these genes, MRPS12 has been widely characterized as a candidate gene for autosomal dominant sensorineural hearing loss DFNA4 18. It has also been found that certain MRP genes are present in candidate regions for retinitis pigmentosa and diseases involving neurological dysfunction (such as Moebius syndrome and Hallervorden-Spatz syndrome) 19, 20.

Spore formation in Saccharomyces cerevisiae is a complex and tightly regulated pathway involving the induction of a large number of genes. Hanlon et al. 21 identified MRPS17 in the cDNA library of spore-specific genes. The homology search showed that the first third of Mrps17 has strong sequence similarity with the bacterial S17 protein, which indicates that Mrps17 is a mitochondrial ribosomal protein. Cells lacking Mrps17 have respiratory defects, and Mrps17-GFP fusion is located in the mitochondria, which also proves the important role of MRPS17 in aerobic circulation. The literature confirmed by Northern blot analysis that MRPS17 and MRPL37 are strongly induced in the middle stage of sporulation, and this induction depends on the presence of intermediate sporulation elements (MSE) in the promoters of these genes. They found that Mrps17 and Mrpl37 are not others. Mitochondrial ribosomal proteins accumulate in the middle of sporulation. These results indicate that Mrps17 and Mrpl37 may have other meiotic-specific effects.

In the present study, we have demonstrated that in comparison with that in normal tissues, the expression of MRPS17 in GC is significantly higher and its high expression is significantly associated with poorer survival of GC patients. Additionally, high expression of MRPS17 has been validated by univariate and multivariate Cox regression analysis to be an independent prognostic factor for GC patients, suggesting that MRPS17 might play vital roles in contributing to occurrence and progression of GC. In order to further elucidate the roles of MRPS17 in GC, we subsequently performed GO and KEGG analysis, revealing that MRPS17-related genes were mainly involved in regulation of cell adhesion and extracellular matrix. Then through immunofluorescence experiment, it was demonstrated that MRPS17, as a MBP, was also detected on cell membrane, suggesting that MRPS17 might promote invasion and metastasis via regulating cell adhesion and extracellular matrix. And, as far as we are concerned, it has not reported by any previous studies that MRPS17 promotes proliferation, invasion, and metastasis of GC. However, it is proven by in-vitro experiments of our study that knocking down of MRPS17 significantly inhibited migration and invasion capability of GC cells. Of course, reduction in migration and invasion capability of GC cells could not be absolutely attributed to knocking down of MRPS17 because other conditions such as senescence, apoptosis and autophagy could also lead to inhibited migration and invasion capability of GC cells. Thus, the relationships between MRPS17 and senescence, apoptosis and autophagy need to be explored by further studies. Then, it was verified by Western blotting that PI3K/AKT signaling pathway was responsible for the promotion of migration and invasion capability of GC cells by MRPS17.

In multiple previously published studies, PI3K/AKT signaling pathway has been reported to play carcinogenic roles in various cancers and to be related with tumor growth and prognosis of patients 22. Fang et al. 23 reported that mutations of genes involved in PI3K/AKT signaling pathway were associated with clinicopathological characteristics of GC patients. And it was also reported that patients with abnormal PI3K/AKT signaling pathway were significantly more likely to experience distant metastases, especially to lung and liver 24, 25. In a study by Wang et al. 26, it was reported that through targeting FAT4 and activating the PI3K/AKT signaling pathway, miR-107 promoted proliferation, migration and invasion of gastric cancer cells, suggesting that PI3K/AKT signaling pathway played vital roles in gastric cancer.

GC is characterized by abnormally enhanced proliferation, migration and invasion of cells, suggesting that ways to suppress proliferation, migration and invasion of cancer cells are fundamental to improving survival of GC patients 27. Many genetic changes lead to abnormal and uncontrolled proliferation, growth, invasion, migration and metastasis of cancer cells 28. As members of the zinc-dependent proteolytic enzyme superfamily, matrix metalloproteinases (MMPs) can be activated by the PI3K/AKT signaling pathway. In human, nearly all the extracellular matrix can be decomposed by activated MMPs. Cancer cells can more easily leave the primary tumor and metastasize to other organs after extracellular matrix has been hydrolyzed by activated MMPs 29.

In the present study, the following aspects were explored to evaluate the roles of MRPS17 in contributing to occurrence and progression of GC: screening overexpressed genes that were significantly associated with prognosis of GC patients, functional verification by Transwell assays, and determination of involved molecular mechanisms. Considering these aforementioned results obtained in the present study, we could draw the conclusion that MRPS17 promotes migration and invasion of GC via the PI3K/AKT signaling pathway and its overexpression in GC is significantly associated with worse prognosis of GC patients, suggesting the potential of MRPS17 as a novel biomarker in treating and stratifying GC patients.

## Supplementary Material

Supplementary tables.

## Figures and Tables

**Figure 3:**
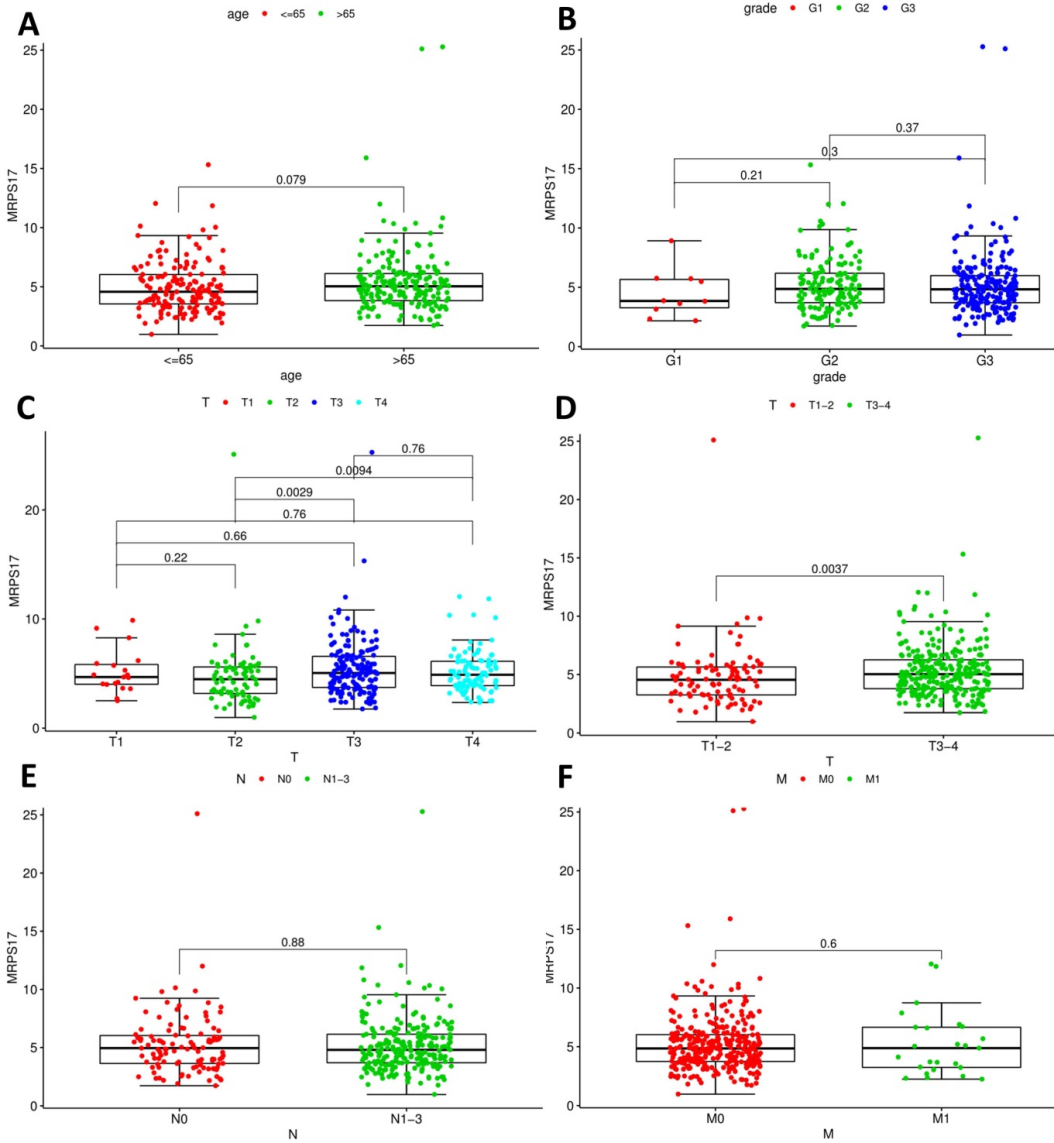
Analyses of correlations between MRPS17 expression and clinicopathological variables in tcga database.

**Figure 7:**
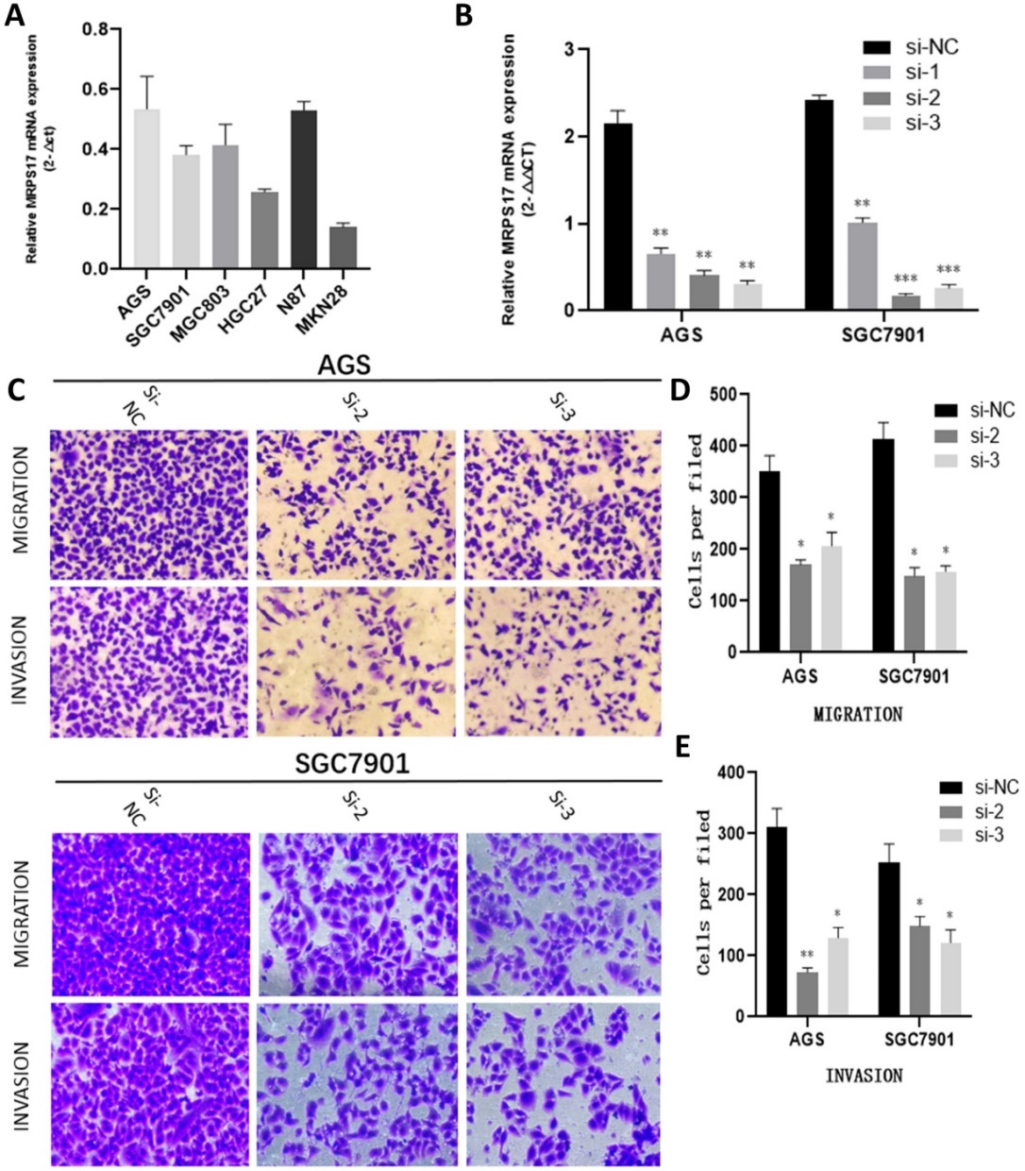
(A) MRPS17 expression levels in AGS, SGC7901, MGC803, N87, HGC27, and MKN28 cells were determined by quantitative real-time PCR analyses. (B) MRPS17 expression levels in MPRS17-silenced cells and scrambled-siRNA-treated cells were evaluated by quantitative real-time PCR analyses. (C-E) Migration (upper C panel and D) and invasion (lower C panel and E) capabilities of AGS and MGC803 cells were assessed by Transwell assays after MRPS17 had been knocked down. Data were demonstrated as mean± standard deviation of triplicate determinations from three independent experiments. Statistical significance was assessed via the unpaired Student's t-test (two-tailed test). *P<0.05, **P<0.01 and ***P<0.001.
