# Supplementary material for: MRPS17 promotes invasion and metastasis through PI3K/AKT signal pathway and could be potential prognostic marker for gastric cancer
Source: J Cancer. 2021 Jun 11;12(16):4849–61. doi: 10.7150/jca.55719 (PMC8247386; doi:10.7150/jca.55719)
Supplement: Supplementary file 1 — Supplementary tables. [file jcav12p4849s1.pdf]

siRNA	The siRNA oligonucleotides
genOFFTM st-h-MRPS17_002	GGCTGAGATCGTTTTCAAA
genOFFTM st-h-MRPS17_003	GGAAGGTGATTGGGACAAA

Supplementary Table 1. The siRNA oligonucleotides against MRPS17.

Characteristic	Mrps17 Positive N=35	Mrps17 Negative N=65
Age		
≤65	26	50
＞65	9	15
Gender		
Female	11	23
Male	24	42
TNM stage		
I-II	15	34
III-IV	20	31
Tumor stage		
T1-T2	4	25
T3-T4	31	40
Lymph node metastasis		
No	30	61
Yes	5	4

Supplementary Table 2. Baseline of immunohistochemistry patients in our center.

Characteristic	N	Characteristic	N
**Age**		**Lymph node**	
≤65	197	N0	132
＞65	241	N1	119
unknow	5	N2	85
**Gender**		N3	88
Female	158	NX	19
Male	285	**metastasis**	
**grade**		M0	391
G1	12	M1	30
G2	159	MX	22
G3	263	**TNM stage**	
GX	9	I	59
**Tumor stage**		II	130
T1	23	III	183
T2	93	IV	44
T3	198	unknow	27
T4	119		
TX	10		

Supplementary Table 3. Clinical baseline data from TCGA database.
